# Supplementary material for: Strong effect of demographic changes on Tuberculosis susceptibility in South Africa
Source: PLOS Glob Public Health. 2024 Jul 23;4(7):e0002643. doi: 10.1371/journal.pgph.0002643 (PMC11265723; doi:10.1371/journal.pgph.0002643)
Supplement: S1 Text — (DOCX) [file pgph.0002643.s003.docx]

**Supplementary Materials:**  Strong Effect of Demographic Changes on Tuberculosis Susceptibility in South Africa

**Authors:** Oshiomah Oyageshio^1†*^, Justin W. Myrick^2†^, Jamie Saayman^3^, Lena van der Westhuizen^3^, Dana R. Al-Hindi^4^, Austin W. Reynolds^5^, Noah Zaitlen^6^, Eileen G. Hoal^3^ ,Caitlin Uren^3,7^, Marlo Möller^3,7*^, Brenna M. Henn^1,2,4*^

**Affiliations:**

1) Center for Population Biology, University of California, Davis, Davis, California, United States of America.

2) UC Davis Genome Center, University of California, Davis, Davis, California, United States of America.

3) DSI-NRF Centre of Excellence for Biomedical Tuberculosis Research; South African Medical Research Council Centre for Tuberculosis Research; Division of Molecular Biology and Human Genetics, Faculty of Medicine and Health Sciences, Stellenbosch University, Cape Town, South Africa.

4) Department of Anthropology, University of California, Davis, Davis, California, United States of America.

5) Department of Microbiology, Immunology, and Genetics, School of Biomedical Sciences, University of North Texas Health Science Center, Fort Worth, Texas, United States of America.

6) Department of Computational Medicine, David Geffen School of Medicine, University of California, Los Angeles, Los Angeles, California, United States of America.

7) Centre for Bioinformatics and Computational Biology, Stellenbosch University, Stellenbosch, South Africa.

† Equal authorship contribution

* Corresponding authors: [bmhenn@ucdavis.edu](mailto:bmhenn@ucdavis.edu), [marlom@sun.ac.za](mailto:marlom@sun.ac.za), [oyageshio@ucdavis.edu](mailto:oyageshio@ucdavis.edu)

Journal: PLoS Global Public Health

This file contains:

Supplementary Methods

Supplementary Discussion

Supplementary Figures A to D

Tables A to D

Supplementary References

**Supplementary Methods**

**Smoking and drinking covariates**

Smoking measures include smoker/non-smoker (“Do you smoke?”), age first started smoking, daily smoking amount, and type (e.g., cigarettes, hand-rolled “zols”, marijuana). Alcohol measures include binary drinker/non-drinker, daily drinking amount, and type of alcohol. Alcohol variables were not included until after the pilot study (November 2018-March 2020). Only the binary drinker/non-drinker and smoker/non-smoker measures were used for statistical analysis because they had the lowest missingness compared to the finer-scale smoking and drinking measures.

**Genetic Data Processing**

DNA samples were collected from saliva with Oragene OGR-500 kits (DNA Genotek) and extracted using prep-IT L2P reagents (DNA Genotek) according to the manufacturer's protocol. Raw genotype data was processed with Illumina's GenomeStudio to call common variants (MAF>0.05), followed by zCall to call rare variants [1]. Bioinformatic pipelines are publicly available via github (<https://github.com/hennlab/snake-SNP_QC>). Prior to genetic ancestry estimation, SNPs out of Hardy-Weinberg equilibrium (--hwe 0.001) and rare alleles (--maf 0.01) were removed from the dataset. The dataset was also pruned for linkage disequilibrium (--indep-pairwise 200 25 0.4). The genotyped data was further cleaned using plink2 [2] with the following parameters: --mind 0.1 --geno 0.05 to remove individuals missing a large number of SNPs and SNPs missing in a large number of study participants, respectively. After cleaning we used the – pca 10 parameter to extract the top 10 principal components (PCs) of the genetic data

**Correcting for Population Structure**

After obtaining the top 10 PCs of our genotyped individuals we included them as covariates in the three logistic regression models we designed (see Main Text).

**PC-corrected Common risk factor model:** TB Status ~ gender + smoking + diabetes + residence + age + SES + PC 1 + PC2 … + PC10

**PC-corrected SES model:** TB Status ~ common risk factor model + age * SES + PC 1 + PC2 … + PC10

**PC-corrected Residence Model:** TB Status ~ common risk factor model + residence * birthplace + PC 1 + PC2 … + PC10

**Random Forest Model**

Similar to logistic regression, random forest is a binary classifier yet differs in that is robust against non-linear associations and unknown interactions [3]. Random forest utilizes a permutation-based approach to generate a hierarchical list of important variables but is unable to quantify the “significance” between an independent and dependent variable. A random forest approach generates a collection of n identically distributed decision trees derived from bootstrapped samples of observations using a recursive partitioning algorithm [4]. This machine learning approach creates splits (daughter nodes) in a tree till a terminal node is reached. At each split, a random subset of *m* predictor variables divides the training sample into two groups with maximal homogeneity. Where *p* represents the total number of predictor variables and *m* is approximately *p*. The algorithm uses approximately two-thirds of all observations to grow each tree and the remaining third, known as the out-of-bag [OOB) sample, is leveraged as validation data to generate a classification error averaged over all trees. This OOB error is calculated for both the original dataset and a randomly permuted dataset. The difference in OOB error between datasets generates a measure of variable importance that is used to rank all the predictors used in the model. The R package *randomForestSRC* [5] was used to implement a random forest model including gender, age, years of education, residence, smoking, and diabetes as predictor variables. The model grew 5000 trees using 5 randomly sampled predictors at each split (mtry) and the minimum size of terminal nodes (node size) set to 10. The values of the mtry and node size parameters were determined using the “tune” function in the *rfsrc* package. A classification table and variable importance metrics were generated from the tuned model. Variable importance was calculated using a permutation-based metric [6].

**Supplementary Discussion**

Despite having the lowest density of individuals living with HIV in South Africa (0 per 5 km^2^] [7], the Northern Cape Province maintains a starkly high TB incidence (645 per 100,00 [8]). One possible explanation is that host genetic factors contribute to risk. Unlike Western African, Asian, and European populations that have lived with *Mycobacterium tuberculosis* for several thousand years [9,10], virulent mycobacterial strains are evolutionarily novel to Southern African indigenous Khoe-San populations. *M.tb* is thought to have emerged in eastern Africa, reflected by the divergent *M. canetti* genome and the basal L5 and L6 lineages (*M*. *africanum*), approximately 2,500-6,000 years ago [11]. From eastern Africa, *M.tb* continued to diversify, spread out of Africa and increase in virulence. Virulent lineages were introduced to southern Africa during the 17th-18th centuries through European colonization and the transport of indentured servants from Asia [12,13]. Under this model, individuals with indigenous southern African ancestry have little prior adaptation to strains of *M.tb*. Even if *M.tb* was introduced to southern Africa via pastoralist migrations 2,000 years ago [14,15], this prehistoric strain may not reflect the virulence of present-day L2 and L4 strains circulating in the region [12,13], leading to an evolutionary mismatch between the host and pathogen. Khoe-San ancestry has been shown to be associated with higher TB risk in the Western Cape [16], however, it is unclear which ancestry-specific genetic mechanisms contribute to potential susceptibility. Here, we show that the highest proportion of ancestry in our Northern Cape population sample is Khoe-San (Fig. 3).

We controlled for population stratification by incorporating genetic PCs to the logistic regression models. We found that our main epidemiological findings from the study remain the same (S1 Data, Fig IV in S1 Text). Namely, male gender and town residence remain important risk factors for active TB progression. Notably PC-correction did not change the observed age by SES interaction effect from the main study but the birthplace by residence interaction effect slightly changes where individuals born in towns and currently residing in rural areas have similar odds of active TB to lifetime rural dwellers (S1 Data, Fig IV in S1 Text).

.

**Supplementary Figures**


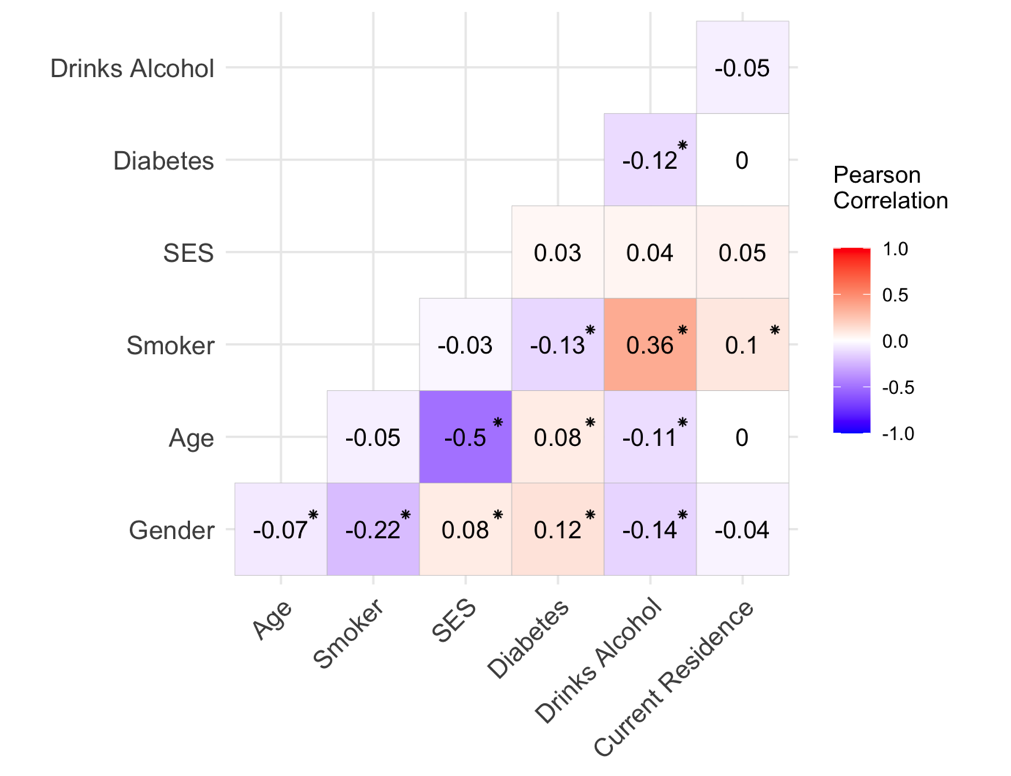


**Fig A. Correlations Among Demographic and Medical Variables for Entire Cohort**. Pearson correlation coefficients were calculated for select demographic, behavioral and medical covariates in our dataset of 774 individuals. Correlations with significant *p*-values (*p*<0.05) are denoted with an asterisk.

**
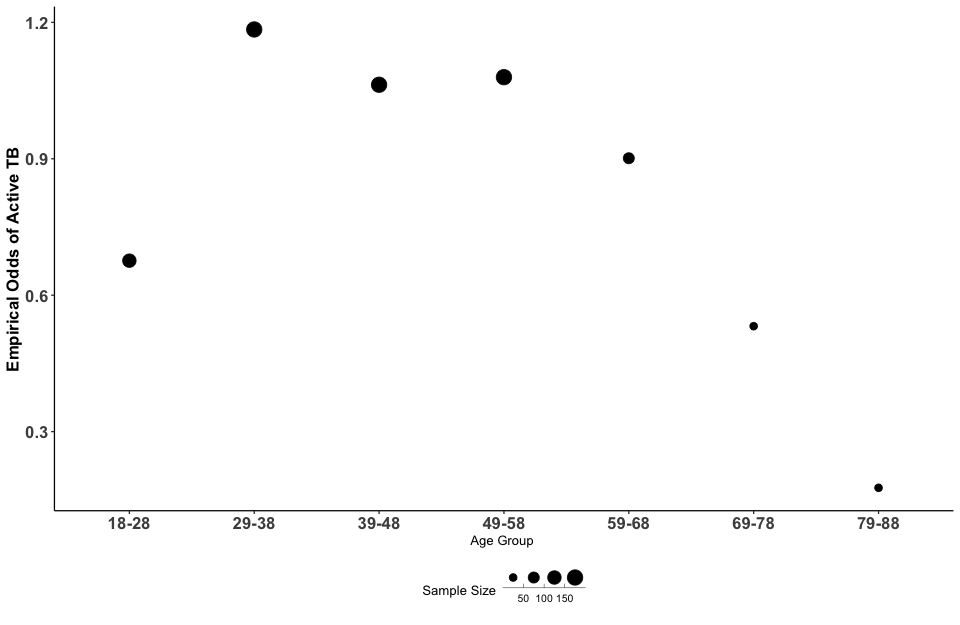
**


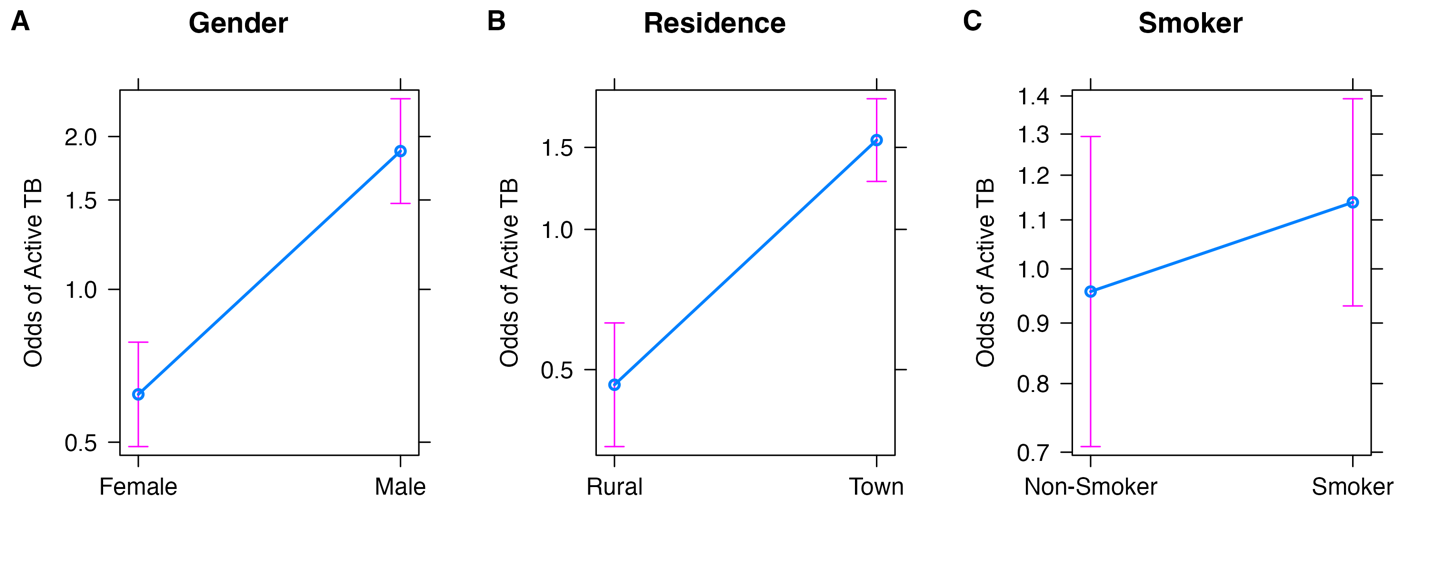
**Fig B.  Empirical odds of active TB by age group**. The x-axis bins our participants into 7 age groups and the y-axis: the empirical odds of active TB. Empirical odds are calculated by dividing the number of controls divided by the number of cases in each age bin. The size of the dots corresponds to the sample size of the age group. The relationship between the odds of progression to active TB and age has an inverted U-shape, in which the oldest cohorts have the lowest odds.

**Fig C. Effect Plots demonstrating the relationship between Active TB Status and A) Gender, B) Current Residence and C) Smoking.** These plots are reported from the best-performing logistic regression model (SES model). Y-axes for all panels show the odds of active TB. We find that the odds of active TB are 3 times higher in Males. Individuals currently residing in Towns have about 3 times higher odds of active TB as compared to individuals currently residing in rural areas. Smoking slightly increases the odds of active TB but is not statistically significant.

**
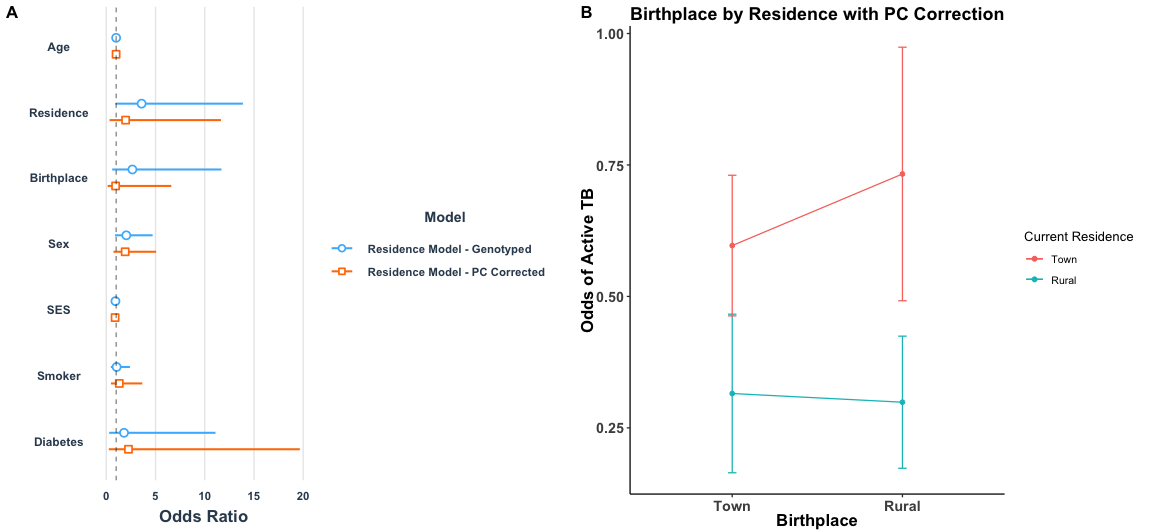
**

**Fig D.** **Correcting for population structure in a subset of the cohort.** Individuals with genome-wide SNP array data (n=159) were reanalyzed to determine whether population structure substantively changed model results. No significant shifts were observed for the common risk factor or SES models. The residence model showed an attenuation of the ‘residence’ and ‘birthplace’ variables. A) Forest plot of the Residence model with (orange) and without PC-correction (blue). The X-axis indicates the odds ratio, while the Y-axis indicates the model variables. B) Effect plot from the residence model with 10 genetic PCs illustrates the interaction term between birthplace residence and current residence. Regardless of birthplace, the odds of active TB is highest in individuals who currently reside in towns.

**Supplementary Tables**

**Table A**. Descriptive Statistics of study variables, stratified by case/control status

| n =774 | variable | Control | Case | test |
| --- | --- | --- | --- | --- |
| Gender | Male | 145 (36%) | 239 (64%) | p <0.0001 |
|  | Female | 255 (64%) | 135 (36%) |  |
| Diabetes | Yes | 357 (93%) | 350 (94%) | 0.6225 |
|  | No | 26 (7%) | 22 (6%) |  |
|  | NA | 17 | 2 |  |
| Smoker | Yes | 241 (60%) | 272 (73%) | 0.0002 |
|  | No | 158 (40%) | 100 (27%) |  |
|  | NA | 1 | 2 |  |
| Drinks Alcohol | Yes | 114 (59%) | 157 (51%) | 0.0901 |
|  | No | 79 (41%) | 149 (49%) |  |
|  | NA | 207 | 68 |  |
| Residence | Rural | 167 (42%) | 70 (19%) | <0.0001 |
|  | Town | 233 (58%) | 304 (81%) |  |
| Age | median | 43.0 | 43.0 | 0.9591 |
|  | mean | 43.6 | 43.1 |  |
|  | sd | 15.7 | 13.2 |  |
| Years of education (SES) | median | 9.0 | 8.0 | 0.0019 |
|  | mean | 8.3 | 7.7 |  |
|  | sd | 3.4 | 3.3 |  |

*Pearson chi-squared tests were conducted for binary variables, while Wilcoxon rank sum tests were used for continuous variables

**Table B**. Odds ratios and *p*-values for the Demographic and Socio-Behavioral Variables used in the Reduced Common Risk Factor & SES Models

| **Reduced Common Risk Factor Model (n = 720)** | | | | |
| --- | --- | --- | --- | --- |
|  | OR | 2.5 % | 97.5 % | p-value |
| **Intercept** | 0.780 | 0.298 | 2.041 | 0.612 |
| **Residence** | 2.930 | 2.044 | 4.237 | p < 0.001** |
| **Gender** | 2.922 | 2.121 | 4.046 | p < 0.001** |
| **Age** | 0.986 | 0.973 | 0.998 | 0.027 |
| **SES** | 0.921 | 0.870 | 0.975 | 0.005* |
| **Smoker** | 1.292 | 0.912 | 1.829 | 0.149 |
| **Diabetes** | 1.341 | 0.681 | 2.640 | 0.393 |
| **Pseudo R²**= 0.18 | | | | |
| **Reduced SES Model (n = 720)** | | | | |
|  | OR | 2.5 % | 97.5 % | p-value |
| **Intercept** | 13.452 | 1.961 | 98.495 | 0.009 |
| **Residence** | 3.018 | 2.098 | 4.384 | p < 0.001** |
| **Gender** | 2.908 | 2.104 | 4.037 | p < 0.001** |
| **Age** | 0.935 | 0.903 | 0.967 | 0.00012 |
| **SES** | 0.666 | 0.542 | 0.812 | p < 0.001** |
| **Smoker** | 1.188 | 0.832 | 1.694 | 0.341 |
| **Diabetes** | 1.288 | 0.653 | 2.538 | 0.462 |
| **Age*SES** | 1.007 | 1.003 | 1.010 | 0.001 |
| **Pseudo R²**= 0.19 | | | | |

**Table C.** Missingness and Imputation Metrics

| **Variable** | **Number missing** | **% missing** | **Imputed** | **Cross Validation Error (%)** |
| --- | --- | --- | --- | --- |
| Alcohol | 310 | 35.3 | yes | N/A |
| Years of Education (SES) | 39 | 4.44 | yes | 2.97 |
| Height | 30 | 3.42 | no | N/A |
| Diabetes | 19 | 2.16 | yes | 9.49 |
| Father’s Ethnicity | 8 | 0.91 | no | N/A |
| Mother’s Ethnicity | 6 | 0.68 | no | N/A |
| Smokes | 3 | 0.34 | yes | 39.11 |

**Supplementary References**

1. Goldstein JI, Crenshaw A, Carey J, Grant GB, Maguire J, Fromer M, et al. zCall: a rare variant caller for array-based genotyping. Bioinformatics. 2012 Oct 1;28(19):2543–5.

2. Chang CC, Chow CC, Tellier LC, Vattikuti S, Purcell SM, Lee JJ. Second-generation PLINK: rising to the challenge of larger and richer datasets. GigaScience. 2015;4:7.

3. Bi Q, Goodman KE, Kaminsky J, Lessler J. What is Machine Learning? A Primer for the Epidemiologist. Am J Epidemiol. 2019 Oct 21

4. Kanerva N, Kontto J, Erkkola M, Nevalainen J, Männistö S. Suitability of random forest analysis for epidemiological research: Exploring sociodemographic and lifestyle-related risk factors of overweight in a cross-sectional design. Scand J Public Health. 2018 Jul;46(5):557–64.

5. Ishwaran H, Kogalur UB. randomForestSRC: Fast Unified Random Forests for Survival, Regression, and Classification (RF-SRC) [Internet]. 2023 [cited 2023 Feb 21]. Available from: https://CRAN.R-project.org/package=randomForestSRC

6. Ishwaran H, Lu M, Kogalur UB. Variable Importance (VIMP) with Subsampling Inference [Internet]. 2021 [cited 2023 Feb 21]. Available from: http://randomforestsrc.org/articles/vimp.html.

7. Kim H, Tanser F, Tomita A, Vandormael A, Cuadros DF. Beyond HIV prevalence: identifying people living with HIV within underserved areas in South Africa. BMJ Glob Health. 2021 Apr 1;6(4):e004089.

8. Kanabus A. Information about Tuberculosis. 2022 [cited 2022 Nov 11]. TB Statistics South Africa. Available from: https://tbfacts.org/tb-statistics-south-africa/

9. Brites D, Gagneux S. Co-evolution of Mycobacterium tuberculosis and Homo sapiens. Immunol Rev. 2015 Mar;264(1):6–24.

10. Comas I, Coscolla M, Luo T, Borrell S, Holt KE, Kato-Maeda M, et al. Out-of-Africa migration and Neolithic coexpansion of Mycobacterium tuberculosis with modern humans. Nat Genet. 2013 Oct;45(10):1176–82.

11. Menardo F, Duchêne S, Brites D, Gagneux S. The molecular clock of Mycobacterium tuberculosis. PLOS Pathog. 2019 Sep 12;15(9):e1008067.

12. Brynildsrud OB, Pepperell CS, Suffys P, Grandjean L, Monteserin J, Debech N, et al. Global expansion of Mycobacterium tuberculosis lineage 4 shaped by colonial migration and local adaptation. Sci Adv. 2018 Oct;4(10):eaat5869.

13. Rutaihwa LK, Menardo F, Stucki D, Gygli SM, Ley SD, Malla B, et al. Multiple Introductions of Mycobacterium tuberculosis Lineage 2–Beijing Into Africa Over Centuries. Front Ecol Evol. 2019 Apr 16;7:112.

14. Henn BM, Gignoux C, Lin AA, Oefner PJ, Shen P, Scozzari R, et al. Y-chromosomal evidence of a pastoralist migration through Tanzania to southern Africa. Proc Natl Acad Sci U S A. 2008 Aug 5;105(31):10693–8.

15. Bos KI, Harkins KM, Herbig A, Coscolla M, Weber N, Comas I, et al. Pre-Columbian mycobacterial genomes reveal seals as a source of New World human tuberculosis. Nature. 2014 Oct;514(7523):494–7.

16. Chimusa ER, Zaitlen N, Daya M, Möller M, Helden PD van, Nicola JM, et al. Genome-wide association study of ancestry-specific TB risk in the South African coloured population. Hum Mol Genet. 2014;23(3):796–809.
