# Supplementary material for: Strong effect of demographic changes on Tuberculosis susceptibility in South Africa
Source: PLOS Glob Public Health. 2024 Jul 23;4(7):e0002643. doi: 10.1371/journal.pgph.0002643 (PMC11265723; doi:10.1371/journal.pgph.0002643)
Supplement: Attachment — Submitted filename: Response to Reviewers.pdf [file pgph.0002643.s004.pdf]

Rebuttal Letter: Strong Effect of Demographic Changes on Tuberculosis Susceptibility in South Africa

Dear PLOS Global Public Health Editor Team,

We were pleased to revise our manuscript entitled “Strong Effect of Demographic Changes on Tuberculosis Susceptibility in South Africa”. Our responses are shown under each comment as bold text. We are appreciative of the insightful comments from both reviewers. We thank you for your patience in our resubmission and look forward to its publication.

Editors Comments:
Both reviewers have major issues related to how ancestry is used to draw conclusions in this analysis; the selection of cases and controls (specifically pertaining to HIV status) and subsequent biases; the validity of methods used to determine TB disease history; and the statistical analyses. Please address all major and minor comments in your revised submission.

Thank you editor. We have now thoroughly reviewed the major issues related to ancestry, case-control criterion, TB disease history and statistical analyses. We respond to each of these concerns in great detail in the subsequent sections

Reviewers' comments:

Reviewer #1: This study focuses on the factors that affect TB risk in an understudied TB endemic region. The authors use a case-control study and find that the following factors increase TB risk: male gender, age x socioeconomic status, and birthplace x residence locality. The variables included in the interactions did not have main effects on TB risk, nor did smoking and alcohol consumption. Finally, the authors performed genotyping and report that participants had a majority Khoe-San ancestry, but genetic ancestry is never linked to TB risk. This paper represents a large amount of collaborative and interdisciplinary work and an important description of an understudied population. My main comment is that several parts of the analyses and decisions behind them are unclear, including whether and how genetic ancestry is analyzed in the context of TB.

Major comments:

The introduction discusses how genetic variation and genetic ancestry can impact TB risk, and line 91 says that ancestry proportions are investigated. However, I didn’t see these analyses anywhere in the paper.
Line 259 – Do you examine ancestry effects on host susceptibility? This concept is brought up again but no analyses are described in the methods.

- **Thank you for pointing out this confusion. To clarify, we are not performing a genetic association study on host TB susceptibility. To limit this confusion we have removed from the introduction the bulk of the text that discusses the host genetic effects on disease susceptibility.**

- **We are leveraging our genotype data to examine ancestry proportions across our clinics. The aim of this was to test for heterogeneity in ancestry among the clinics in our dataset. The results from this analysis are found in the Results section "Genetic ancestry and ethnicity".**

Are there any relatives in your study? Or population structure? If so, please consider linear mixed effects models that control for pairwise genetic relatedness and/or principal components of genetic variation. Since you have genetic data, I'm not sure why it isn't used for data analysis either to allow for modeling of a key covariate and/or to examine ancestry effects on susceptibility.

- **We do see moderate population structure across clinics. Please see Supplementary section "Genetic ancestry and ethnicity" for detailed discussion.**
- **We agree it would be appropriate to include PC covariates or GRM for this analysis. It is the goal of this project is to perform these association analyses in the future with more data, however, we have only genotyped ~159 samples, which will be underpowered to identify genetic associations. Grant applications to genotype the full dataset have not yet been funded and would total ~$60,000 prohibiting us from including them in the current paper.**

Why are HIV positive individuals not considered cases? They still have TB. From the flow diagram in Figure 1 it also looks like HIV positive status does not exclude someone from being a control, which seems like it would create bias if they are excluded from one group and not the other.

- **We are not including people living with HIV (PLWHIV) who are coinfected with TB because this study's focus is on the socio-behavioral factors that affect people's risk of progressing to active TB. HIV increases TB risk by 20-fold, the largest known risk factor for progression to active TB and would attenuate our understanding of socio-behavioral TB risk factors**
- **We originally kept PLWHIV controls in the study as we believed they were "resisters"/"early clearers" see (Kroon et al., 2020; Verrall et al., 2014).**
- **That said, we have taken the reviewers' suggestion and removed PLWHIV controls from the analysis so that controls and cases have the same HIV inclusion criteria (n=774).**
- **We adjusted the flow diagram of our study design (Figure 1) to reflect this change.**

Minor comments:

Line 52 – "The extent of these determinants' effects varies across and within populations, necessitating epidemiological studies in differing contexts and communities". I'm sure this is true, but a few examples would really help. In general, the motivation for the particular study (beyond studying an understudied population) could use some fleshing out.

- **We have included examples of the variation referenced in line 52 and expanded this section of the introduction to give better examples of TB risk factors generally and in South Africa; the text is pasted below:**

- **"Determinants of active TB progression are multifaceted, including: genetics, nutrition, social and economic conditions, behavior, and sex-specific biology (1,7,8). Initial *M.tb* infection is largely determined by exogenous factors, such as TB prevalence in the community, population density (e.g., prisons), and working conditions (e.g., mining, healthcare workers) (9–13). The lifetime risk of progressing to active TB following infection is 10%. This risk is the highest within the first 5 years of initial infection and is typically considered to be mediated by the host's innate and cell-mediated immune system (9,14). Individual (i.e., host) factors, however, have also been shown to increase risk of progressing to active disease. These include HIV/AIDS, poor nutrition or low body mass index, indoor air pollution (e.g., cooking with wood and poor ventilation, smoking, alcohol abuse, diabetes mellitus, and intravenous drug use (1,9,14,15). Studies in India have shown undernutrition to be among the strongest determinants for TB risk (15). In South Africa specifically, poor living conditions, unemployment, low SES, age and male gender, race, smoking, and marital status have all been identified as contributing to TB risk (16–21).The extent of these determinants' effects can vary across and within populations, necessitating epidemiological studies in differing contexts and communities (8). Compared to medium or high-TB-incidence countries, the effect sizes for alcohol abuse, homelessness, and intravenous drug use are stronger in low-incidence populations (22) . In South Africa, multilevel modeling approaches have shown that provincial (16) and community income inequality (18) have strong effects on TB incidence and progression, independent of individual-level risk factors."**

- **We have also revised the introduction to provide more clarity on the motivations of this study, please see the quoted text below from the last paragraph of the introduction :**

  - **"Here, we present a TB case-control study characterizing the individual-level risk factors for TB progression among HIV-negative patients with suspected TB from the Northern Cape. Because the Northern Cape has the highest TB incidence but the lowest HIV prevalence and PLWHIV density, and overall low population density, canonical risk factors do not appear to be driving the extraordinary incidence rates. To focus on factors other than immune suppression, we exclude PLWHIV from the analysis. We test three separate models comprising common risk factors, as well as factors that may uniquely affect South Africa. We find that exogenous factors like SES, cohort age, and residence/birthplace have a strong effect on TB progression, often equal to or greater than endogenous factors like gender or smoking/alcohol. These**

> **results suggest further research into the causal mechanisms behind exogenous risk factors and opportunities for TB prevention are warranted."**

Can you provide some more background about why TB/HIV co-infection is so common? Is the thought that individuals succumb to TB after being infected with HIV because of compromised immune systems? It is also confusing that the intro focuses on TB/HIV co-infection, and then these individuals are excluded from the actual study.

- **We agree about this confusion and we have revised this section in the introduction to clarify the motivation of excluding PLWH from the study while also acknowledging the immunological impact of HIV on TB progression. HIV is the leading risk factor for TB, however, a majority of people with TB are not co-infected with HIV. We are focused on studying the latter population, however, we do not want to disregard the major impact of HIV on TB progression and transmission. Below is the revised text from the introduction:**
- **"HIV increases TB risk by 20-fold, the largest known risk factor for progression to active TB, and TB is the leading cause of AIDS-related deaths (23). The effect of HIV on suppressing the host immune system can reactivate a latent *M.tb* infection and increase susceptibility to initial infection (14,23,24). Despite HIV being the strongest TB determinant, other TB risk factors explain the majority of global TB cases (9). In South Africa, 59% of people with TB on the National TB Programme (i.e. on TB medication) are co-infected with HIV (25). However, South Africa's first national TB prevalence survey found that only 28% of people with TB were also people living with HIV (PLWHIV) (25), a finding underscoring the necessity to extend TB research to those living without HIV in high TB burden areas. At the provincial level in South Africa, HIV prevalence explains little of TB incidence ($r^2 = 0.036$) (26). The Western and Northern Cape Provinces have among the highest TB incidence yet the lowest HIV prevalence (27)."**

It is very inconvenient to have the figure legends embedded in the text, but the figures themselves at the end of the manuscript.

- **We agree with the reviewer and we were required by PLoS to arrange the manuscript as such as shown here : https://journals.plos.org/globalpublichealth/s/submission-guidelines**

It's not clear until the methods that the study is really comparing suspected/latent TB individuals vs individuals with currently active or past active TB. Could you make that clear in the intro and reframe some of the background about risk factors for progression to active TB (rather than risk factors for TB alone)?

- **We have revised the introduction to incorporate these suggestions, including clarifying our cases as people with active TB (i.e., disease) and controls as exposed to TB &/or latent TB (LTBI, infected with mycobacterium, which hasn't developed into disease, that is TB).**

Throughout the introduction we revised our wording to specify risk factors for active TB or TB progression. Please see below for the revised text clarifying our cases and controls in the introduction:

- **"In case-control studies, controls should have similar disease exposure profiles to the cases. Population-based controls risk differential disease exposure, a concern in low-incidence populations that can bias statistical associations. However, in high-incidence populations with well-characterized disease burdens and transmission, population controls can greatly improve statistical power (28). In South Africa, TB transmission is driven largely by community spread rather than household contacts (29,30) and TB latency increases with age, reaching 80% by age 30 (31–34). These epidemiological conditions help ensure our cases and controls have approximate disease exposure profiles. In this context, controls from our study sample are people with suspected TB from local health clinics who were microbiologically confirmed to not have active TB. Controls are assumed to either have been previously exposed to or infected with M.tb (i.e., LTBI). Cases, in contrast, are people who have microbiologically confirmed active TB or self-report a past active TB episode."**

Line 159-161 – Please expand on this procedure. How were Google Earth images translated into population size? It would also be nice to compare censuses data to your Google Earth method for a few regions that have census data, to see how correlated the methods are and if there is any bias. Also, this part of the methods makes it sound like you gather population density info, but then there's not explanation of how this is binarized into the variable you use for modeling (town vs rural).

- **Added to Methods: "Places that didn't have a census size available in Stats SA (statssa.gov.za) typically were very small communities, like a farm or a small settlement. By visualizing the settlement through Google Earth, we were able to estimate whether the community size was >2000 estimated people. We used the average household size from the census data from all locations in the district listed by Stats SA (statssa.gov.za), then counted the number of dwellings in blocks and multiplied by the average household size (4) to get an estimated population size. While an imprecise measure of population size, the lack of government census data for a community is itself an indicator of its rural locality."**
- **For example, one location for which there's no census data is shown below, located in the Mier Local Municipality, Northern Cape Province. We took the average household size for all the towns in the Mier Local Municipality for which there is census data [] and multiplied it by the number of dwellings we estimated for Eenduin from Google Earth. Below is a screenshot of Andriesvale.**

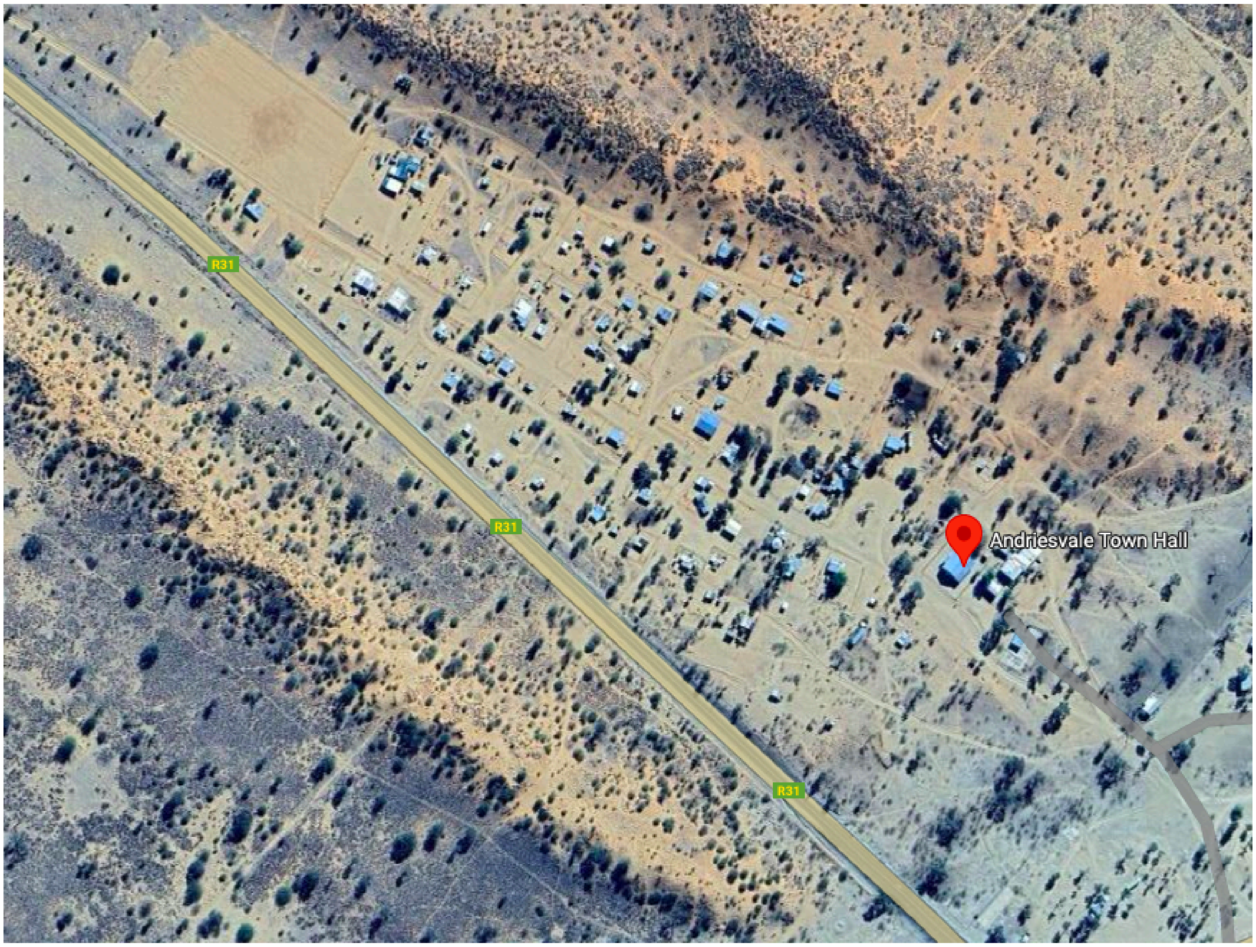

- **And a close-up of a set of parcels with a red square around what is considered a "household/dwelling"**

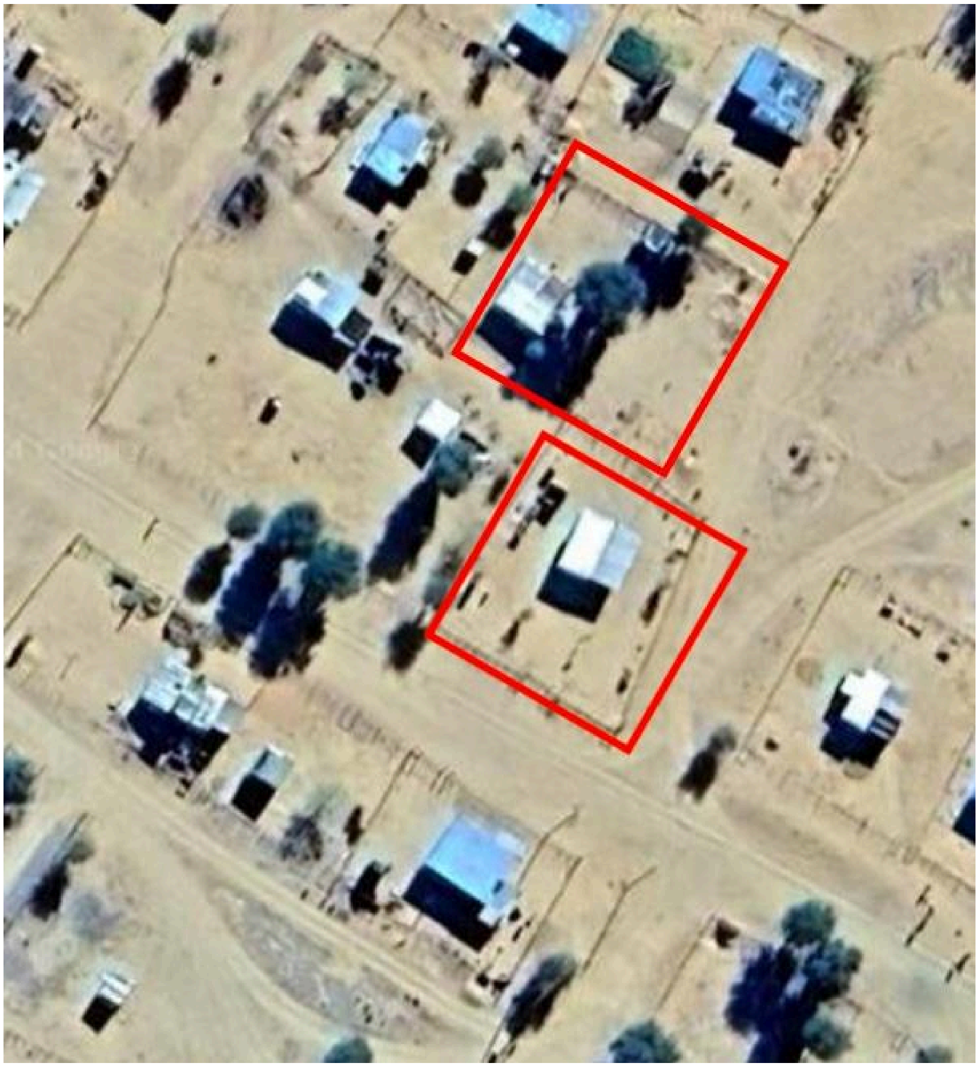

- **We revised the wording to clarify that we are measuring population size and not population density. Here is the revised text in the Methods, "Residence and birthplace locations are categorized according to population size, rural (≤2000 people) and town (>2,000 people)."**
- **In order to binarize this variable, we plotted a rough histogram of the town/settlement size. Based on the values shown, there was clearly a dropoff in settlements greater than 2,000. For this reason, they were classified into two categories.**

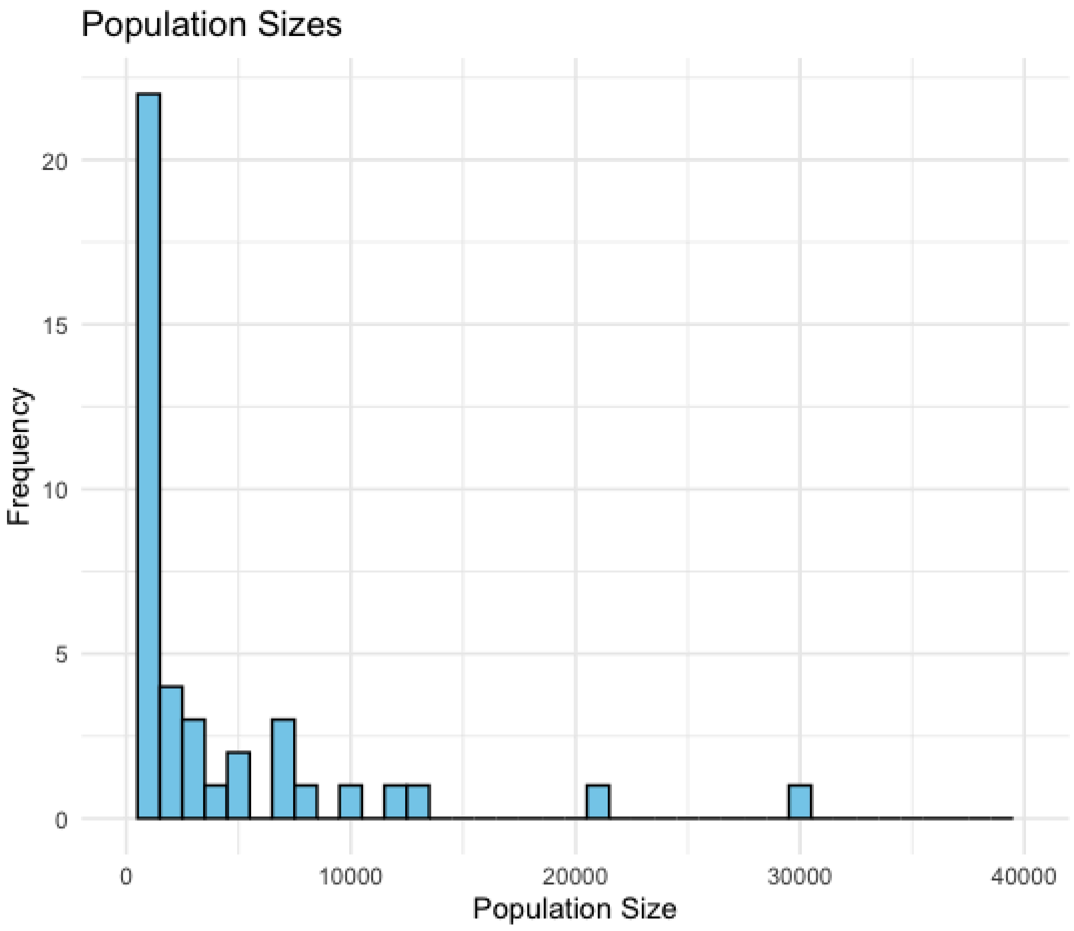

Line 219 - Please justify the use of the particular populations included as reference groups in the ADMIXTURE analyses. Also why was k=5 chosen? Were other values of k tested?

- **We have previously analyzed genetic ancestry data for other populations from the Western and Northern Cape (e.g. Uren et al 2016, Genetics; Petersen et al. 2013, PLoS Genetics; De Wit et al 2010, Human Genetics). Based on the observation of ancestry proportions we know that there are two ancestries (eastern Asian: Indonesian, Malay, Chinese; southern Asian: Sri Lanka, India, Bangladesh) which are minority ancestries typically less than 5% derived from the VOC slave trade at lower fractions compared to Cape Town. European migration is well documented historically and in interviews. Interviews also suggest that recent migration from Bantu-speakers impacts the communities, including Xhosa, Tswana and others. Finally, the Khoe-San ancestry could have been broken down into 2 clusters based on some differentiation between Khoekhoe and San-derived demographic histories. But this ancestry differentiation estimate tends to be unstable in ADMIXTURE, likely due to small samples sizes for the Khoekhoe. Hence, k=5.**

Line 290 – Why not run the common risk factor model for the individuals included in the

residence model? That way you could ask whether the inclusion of residence variables improves the model. As it stands, it's very unclear because there is no base model to compare to.

Thank you for this suggestion; we have re-analyzed the data as suggested using the same group of individuals across models. The "adjusted" common risk factor and "adjusted" SES models (n=720) are available in Table C in S1 Text. The adjusted common risk factor model had a pseudo r2 of 0.18 ,the adjusted SES model pseudo r2 of 0.19 (Table C in S1 Text) while the residence model had a pseudo r2 of 0.19. Effect sizes and significant variables remain consistent across the models with 774 vs 720 individuals. We still report the model results of n =774 in Table 1 of the main text and Table B in S1 Text.

Table 1 – You don't need a blank row for "Current Residence - Rural (reference)"

Thank you. We have removed the blank row for "Current Residence - Rural (reference)

Apologies if I missed it, but could you provide some ideas in the discussion about the interaction between birthplace and current residence, since the effects go in the opposite direction of what was expected?

- **Thank you for the suggestion. We have clarified our hypothesis regarding town and rural birthplace effect in the Results section (under Hypotheses...) as below:**
    - **"Residing in an urban or rural environment is an established risk factor for TB status (11,55–57) and was included in our common risk factor model, as above. Previously, we have shown that migration from an individual's natal town has increased over the past two generations in the rural Northern Cape Province (39). In addition, a recent longitudinal study leveraging data from South Africa's National Health Laboratory Service showed that incorporating cross-municipality migration improves the ability to predict TB incidence (58). For our "residence model", we hypothesized that migrating from a rural to urban area in one's lifetime increases the odds of active TB status due to greater exposure to M.tb. Here, we include an interaction between current residence and birthplace in the common risk factor model. Setting this interaction allows us to examine four patterns, namely: lifetime rural residence, rural birthplace to urban residence, urban birthplace to rural residence, and lifetime urban residence."**
- **We provide ideas in the Discussion about this unexpected finding. To summarize: Urban birthplace to rural residence is the most protective combination. We agree that this is somewhat surprising given the strong effect that current residence in town has on TB in the model. We hypothesize that those born in towns who later moved to a rural area benefit from BCG vaccination during childhood and/or improved access to nutrition compared to rural**

birthplace. Subsequently living as an adult in a rural area may decrease adult exposure to TB. The combination of these factors, speculatively, may result in this relatively lower odds of active TB in individuals in the rural-residing, town-born group compared to the other groups in the birthplace by residence interaction effect.

Reviewer #2: This study aims to assess risk factors for TB in a sample of patients attending health facilities in the Northern Cape province of South Africa. Consistent with previous studies, male sex and lower educational attainment were found to be risk factors, but in further analysis the effect of educational attainment was found to differ substantially with respect to age. Also somewhat surprisingly, although there was a substantially higher TB risk in town dwellers, people who had been born in towns and resided in rural areas had the lowest TB risk.

Major comments

The study design is unusual because the “cases” are defined as people who either have current TB (microbiologically confirmed by GeneXpert testing) or who self-report having had TB previously. The latter is less ideal because self-report is generally not considered very reliable (there may be recall bias, particularly if the TB episode occurred a very long time ago, or there may be social desirability bias given the stigma associated with TB). The authors don’t report what fraction of the cases were self-reported versus microbiologically diagnosed, and so it’s difficult to know how serious this limitation is. This information is also important in assessing how much of the TB is past versus current TB. Did the authors assess whether there were differences in the TB risk factors when considering separately the current TB cases and the self-reported past TB cases?

- **We are aware of the potential for recall bias, and point out this limitation in the discussion. The statistical impact of recall bias would likely result in the attenuation of our effect sizes rather than producing spurious results. We also face this issue with our controls as well; people may not remember that they had TB in the past, which would also attenuate our effect sizes.**
- **JWM (a trained anthropologist) performed qualitative research in this population/community to investigate the biases the reviewer aptly points out in our self-report TB data. In this community TB is not stigmatized, at least not to the degree that HIV is. People are generally open to talking about TB, their own TB history, and TB in the community. There is widespread understanding of how TB is medically treated and the governmental subsidies you receive while on TB treatment. Additionally, we employed community members as research assistants, which are more like peers compared to nurses, and we find that participants are more willing to disclose their TB history, smoking and drinking behavior. We do find a social desirability effect when nurses collect smoking data, for example, compared to our research assistants. Additionally, because TB treatment is 6 months long, and those who do not complete treatment could relapse or develop life-threatening drug resistant TB, it is a life event that people generally remember. Anecdotally, a participant claimed to never have had**

TB, but her mother was nearby and corrected her, she had had TB as a child. Again, this type of recall bias is inherent to self-report data and will likely introduce statistical noise rather than driving spurious results.

- **We clarified the fraction of the cases that are self-reported versus microbiologically confirmed (see "Case-Control Assignment"). Out of the 374 cases, 166 are self-reported past TB, while 208 individuals were clinically diagnosed.**
- **We re-ran the common risk factor models separately for the microbiologically confirmed TB cases and the self-reported past TB cases; our original results did not change.**
- **Briefly; Male Gender and Town Residence remain the significant risk factors for active TB across all models. SES was significant in the Self-report case only model but not Clinically confirmed TB model. All other variables were not significant in both models. Below we report the odds ratios for Gender, SES and Residence**
- **Clinically confirmed TB common risk factor model - Gender: (OR = 4.06[2.75 -6.05] , p < 0.001), Residence: (OR= 7.08 (4.25 12.3), p<0.001) , SES: (OR= 0.92 [08.6-0.99], p =0.036)**
- **Self-report case only common risk factor model - Gender (OR= 2.23[1.51-3.29],p < 0.001),Residence:(OR= 1.78 [1.20-2.69], p <0.001), SES:(OR= 0.90 [0.83-0.95], p< 0.0005)**

There are a number of places in the paper where the authors allude to historical changes (or in some cases "demographic changes") as possible explanations for interactions between risk factors, but without providing coherent explanations or clear hypotheses. For example, in lines 377-9: "This study demonstrates a possible unique historical context to South Africa, (post-)Apartheid differential effects between sociodemographic and health outcomes." It is difficult to make a convincing argument when past TB and current TB are analyzed as a single outcome.

- **As mentioned above we reanalyzed our models separately for self-reported past TB and current clinically past confirmed TB, and the results do not change.**
- **With the clinically confirmed analyses the age by SES interaction was [OR=1.01 [1.00,1.01, p =0.0091) while for the self-reported past tb [OR = 1.018 [1.004, 1.014] , p < 0.001]**
- **The "U-shaped" effect remains when past TB and current TB are analyzed as separate outcomes.**

Related to the previous point, the authors could do more to compare their study with other South African studies on the role of socio-economic status. It's difficult to know whether the risk factors they describe as "different from other global populations" (in the abstract) are really a peculiarity of this study design/setting or consistent with other South African data. As examples of South African studies that have assessed the effect of socio-economic status on TB risk, see Ncayiyana et al (BMC Infectious Diseases, 2016, 16:661), Mahomed et al (Int J Tuberc Lung Dis, 2011, 15(3):331-6) and Harling et al (Social Science & Medicine, 2008, 492-505).

- **Thank you for your comments. We have rephrased the Abstract and included these citations in the Introduction with the goal of providing more specific context for our study in South Africa.**
- **Abstract: "We also tested for demographic factors that may uniquely reflect historical changes in health conditions in South Africa."**
- **Introduction: . " In South Africa specifically, poor living conditions, unemployment, low SES, age and male gender, race, smoking, and marital status have all been identified as contributing to TB risk (16–21)"**

It should also be acknowledged that educational attainment may be a crude measure of socio-economic status (especially in the post-Apartheid context of universal access to education). In addition, participants were recruited from public health facilities, and so people using private healthcare are not represented, which obviously limits the ability to draw conclusions about TB risk at higher SES levels.

- **Thank you for your comments. We have noted the limitations of educational attainment as a crude measure of SES in the discussion. Please see below the revised text:**
- **"Highest completed level of education (e.g., grade, diploma, degree, etc.) is a blunt measure of SES, and does not fully capture all SES facets, including social, economic, and cultural capital (63,64), and universal access to education increased post-Apartheid (65). Additionally, we only sample from community health clinics, not private clinics, thereby missing a fraction of the SES spectrum. "**
- **We understand that only recruiting from public health facilities could be a source of selection bias and we included this as a limitation in our discussion. "According to the National TB Prevalence survey of South Africa, only 8.1% of symptomatic patients sought care at private healthcare clinics. In our manuscript we state: "Community health clinics are the front line for TB screening and treatment, visited by 87% of people who seek TB care (South African National Department of Health et al., 2018)."**

The finding that TB risk is lowest in people who were born in towns but now reside in rural areas is a bit unexpected. The authors attempt to provide an explanation for this is the Discussion, arguing that BCG vaccine coverage is probably better in urban areas than in rural areas, meaning the town-born individuals are more protected early in life. But this argument seems a bit tenuous, given that the authors don't cite any evidence of low BCG vaccine coverage in rural areas, and given that the BCG vaccine is generally thought to provide only limited protection in early childhood (a life stage that most people are unlikely to remember when asked if they've ever had TB). Another explanation might be that the people who were born in towns but now reside in rural areas are generally of higher socio-economic status.

- **Thank you for your comment. We have revised our discussion section to flesh out the unexpected result that TB risk is lowest in people who were born in towns but now reside in rural areas. The BCG vaccine hypothesis was posed by the clinic staff when we were returning results, as such, we revised the language to make this more clear and include the reviewer's valid critiques. We feel it's important to include feedback on the study from the community. We also agree that SES may also play a role.**
- **Discussion excerpt here: "Unexpectedly, the individuals with the lowest odds of active TB are those born in a town who move to a rural area. Those born in a larger town who move to a rural area may benefit from a greater likelihood of BCG vaccine and low adult M.tb exposure in combination with higher SES. When these results were returned to the community, the clinic staff hypothesized that despite nationally standardized BCG vaccinations, rural areas may have lower vaccination rates (observation communicated by clinical staff in the study catchment), however, the benefits of the BCG vaccine attenuate in adolescents and memory of childhood TB episodes suffer from recall bias. It is possible that town-born rural-residence group accrued more wealth in towns before moving to a rural area, affording a different lifestyle than their rural neighbors (e.g., afford larger homes, less crowding, cleaner cooking fuel, etc.). This unique combination of factors may explain why the town-born rural-residence group has even lower odds of active TB compared to the lifetime rural dwellers. Future work should consider collecting birthplace in addition to current residence to better identify TB risk. "**

The decision to exclude people living with HIV (PLWH) from the cases but not the controls seems a bit odd, and the rationale isn't clear in the main paper. In the supplementary materials it becomes clear that the authors are trying to focus on non-HIV risk factors (it would perhaps have helped to explain this in the main text). But if one is going to exclude PLWH, does it not make sense to exclude them from both the cases and the controls? Describing HIV-positive non-TB patients as "resisters" (in the supplementary materials) is a bit weak, since the authors haven't done any TST/IGRA testing, but even if it is true, how does this justify the decision to include them as controls?

- **We agree that the bias posed by having different HIV criterias for cases and controls is not mitigated by our biological argument for including them and treating them as TB "resistors" (i.e., people infected with TB (latently) and clearing the disease or not progressing to active TB despite being immunological compromised by HIV). We therefore have applied the same HIV inclusion criteria for cases and controls and reran all our statistical analyses without PLWH TB controls (and without PLWH TB cases).**
- **We also revised our introduction to make it clear that we are focusing on non-HIV risk factors, please see excerpt from the introduction:**
    - **"HIV increases TB risk by 20-fold, the largest known risk factor for progression to active TB, and TB is the leading cause of AIDS-related deaths (23). The effect of HIV**

on suppressing the host immune system can reactivate a latent M.tb infection and increase susceptibility to initial infection (14,23,24). Despite HIV being the strongest TB determinant, other TB risk factors explain the majority of global TB cases (9). In South Africa, 59% of people with TB on the National TB Programme (i.e. on TB medication) are co-infected with HIV (25). However, South Africa's first national TB prevalence survey found that only 28% of people with TB were also people living with HIV (PLWHIV) (25), a finding underscoring the necessity to extend TB research to those living without HIV in high TB burden areas. At the provincial level in South Africa, HIV prevalence explains little of TB incidence (r2 = 0.036) (26). The Western and Northern Cape Provinces have among the highest TB incidence yet the lowest HIV prevalence (27)... "Here, we present a TB case-control study characterizing the individual-level risk factors for TB progression among HIV-negative patients with suspected TB from the Northern Cape."

The interpretation of Figure 2 could be questioned. The authors interpret the (relatively) low TB risk at older ages as indicating a "survivor bias". Although that is possible, one should also consider that (a) these are self-reported data that could be affected by recall bias (more important in older adults), and (b) these are data from patients attending health facilities, and not a population-based sample (one might expect that older adults would be more likely to be attending health facilities because of chronic conditions). I did also wonder whether it was really necessary to include Figure 2 in the paper (and if so, whether it should not go in the Results section, rather than the Methods section).

- **We reanalyzed our data grouping cases into self-report and microbiologically confirmed cases and we find that the low odds for TB at older ages still holds for both groups. Although recall bias is always a concern in self-report data, we do not see a strong indication of it in this case.**
- **We may be oversampling older individuals due to point B, however, our average age is 43 years old (please see age distribution below) and is normally distributed; Shapiro-Wilk normality test: W = 0.9806, p-value = 1.308e-08**

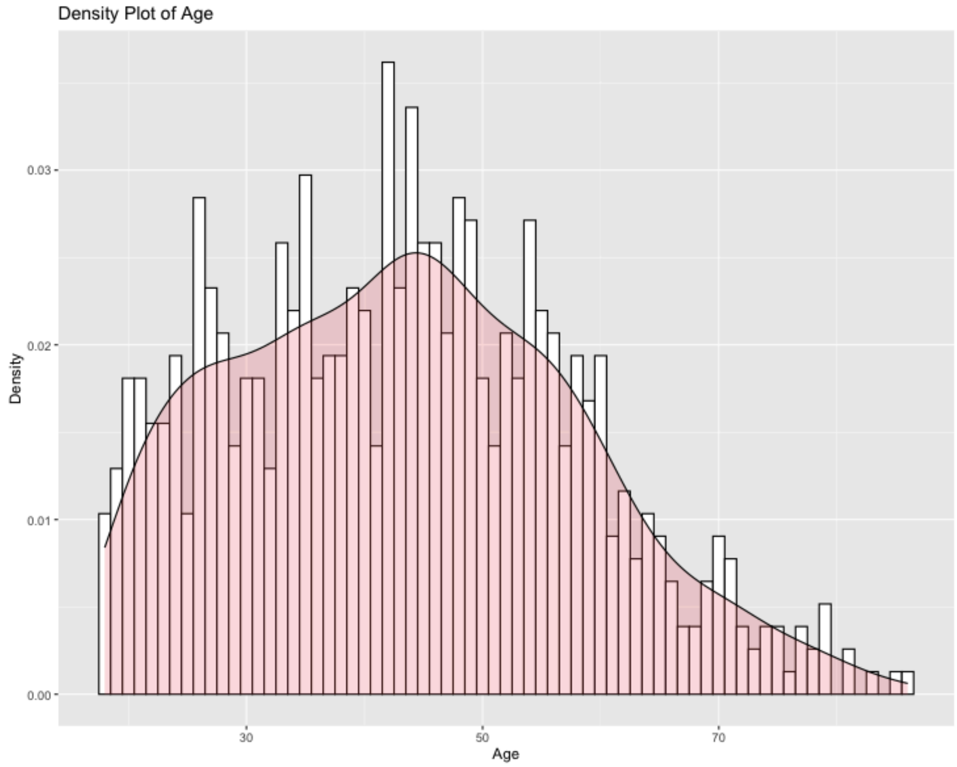

-
- **We have adjusted the language in our manuscript so that survivor bias is explained as a possibility and not a certainty. In the results we state " A form of survivor bias, in which relatively fewer of the oldest adults present as cases because of earlier mortality due to TB, could explain this pattern". In the Discussion we state "This drop in empirical odds after age 58 is most likely due to the mortality of individuals with TB, potentially a signal of survivor bias (Swanson et al., 2018)."**
- **We decided to leave Figure 2 in the manuscript, but have moved it to results rather than the Methods section as you suggested.**

A limitation is that 35% of the alcohol data in this study were imputed. Although this is mentioned in the supplementary materials, **it's important enough that it should be mentioned in the main text.** The high level of missing alcohol data could well explain why alcohol wasn't found to be a significant predictor of TB risk (in contrast to most other studies, which show a strong association between heavy alcohol use and TB). Another concern regarding the imputation is that it seems the authors are using only one imputed dataset (?). Normally one would generate multiple datasets and then combine the results using Rubin's rules.

- **Thank you for your comment. Indeed, the high missingness of alcohol is a major limitation; we have removed alcohol as a variable from all our models.**
- **We have now used Rubin's rules to pool our two imputed datasets (See Methods)**
- **For the purposes of visualization of model covariate effects we used only one imputed dataset. This is because the model object produced by MICE and psfmi are incompatible with the**

standard R packages used for visualization such as "effects". Additionally there were no stark differences between the pooled ORs from Rubin's rules and the OR's from running the models on each imputed dataset individually.

The study includes ancestry data based on genetic testing in a subset of the patients. I'm not an expert on this, but it did feel a bit like this had been "tacked on" to the paper and didn't really fit with the rest of the paper, nor was it clear why it had been included. Due to the small number of patients with ancestry data, the authors did not explore whether ancestry was related to TB risk. There was no mention of the relevance of the ancestry data in the Discussion (although there was some generic discussion on the potential role of genetics in the supplementary materials). As such, it felt as if the ancestry data could have been dropped from the paper without changing any of the conclusions. There also isn't enough information on how the genetic data were analysed (lines 213-225), which makes it hard for the uninformed reader to understand Figure 3 when it is presented later. (Specifically, how are the proportions in Figure 3 calculated? Is each individual categorized according to the ancestry pattern that is most common in the individual, or is this an average of the ancestry proportions across all individuals? And what do the boxes and lines in panel A represent?)

- **We are leveraging our genotype data to examine ancestry proportions across our clinics. The aim of this was to test for heterogeneity in ancestry among the clinics in our dataset. The results from this analysis are found in the Results section "Genetic ancestry and ethnicity", which we have moved into the main text. We have also added a discussion paragraph (below):**
- **"Finally, we highlight that our study included enrollment from 12 different clinics, some of which are more than 250 kilometers apart. We surveyed ethnicity and genetic ancestry in order test for population structure in the sample. Such structure can confound analysis if genetic ancestry tracks differential host risk for progression to TB or if different ethnicities have different cultural norms. Previous studies have described the high proportion of Khoe-San ancestry in Northern Cape communities but these largely focused on descendant groups who identify as Khoe-San (e.g. the ≠Khomani San, the Nama, Karretjie) rather than the general population (81). Here, we show the clinical study population to be admixed with 4 other distinct ancestries (Fig 3), demonstrative of recent historical events. These include the Bantu expansion into Southern Africa, European colonialism, the Dutch East India Company (aka VOC) slave trade, and the displacement and forced settlement of indigenous South African Khoe-San groups, especially in the last few generations in the Northern Cape (50,82). We do find significant genetic heterogeneity across clinics. To our knowledge, this is the first study to report ancestry proportions of clinical populations in the Northern Cape Province, South Africa. This work provides a baseline to design well-informed future studies, such as exploring host genetic correlates of active TB progression in this population (Supplementary Discussion in S1 text)."**

- **Since we have only genotyped ~159 samples we are underpowered to identify genetic associations. Grant applications to genotype the full dataset have not yet been funded and would total ~$60,000 prohibiting us from including them in the current paper.**
- **The proportions in Fig. 3 are the average ancestry proportions across all individuals at each clinic. We have revised the text in the caption to make this more clear, please see the excerpt below:**
- **"A subset of participants (n=159) was genotyped for preliminary ancestry analysis to obtain the average ancestry proportions across all individuals for each clinic ."**

Minor comments

The title of the paper refers to an "effect of demographic changes", which sounds very vague (and even after reading the abstract it's not quite clear what the authors means by "demographic changes"). I would recommend trying to be more specific.

- **We appreciate the inclination to make the title more specific. Unfortunately, we find it difficult to include the specific SES and residence/birthplace results in the abstract while still being pithy. We have clarified in the abstract what we mean by demographic changes, please see the abstract text selection below:**
  - **"We also tested for demographic factors that may uniquely reflect historical changes in health conditions in South Africa. We find that socioeconomic status (SES) significantly interacts with an individual's age (p=0.0005) indicating that protective effect of higher SES changed across age cohorts. We further find that being born in a rural area and moving to a town strongly increases TB risk, while town birthplace and current rural residence is protective."**

In the final sentence of the abstract the authors refer to this as a case-control study. This should have been made clearer earlier on in the methods section of the abstract (and the authors should have included the numbers of cases and controls in the results section of the abstract).

Thank you for this suggestion. We have modified the abstract as below: "We leveraged the population's high TB incidence and community transmission to design a case-control study with population-based controls, reflecting similar mechanisms of exposure between the groups. We recruited 1,126 participants with suspected TB from 12 community health clinics and generated a cohort of 774 individuals (cases =374, controls =400) after implementing our enrollment criteria."

The explanation of the interaction terms in the abstract is very unclear. What does the OR of 3.05 actually represent – which group is being compared to which? Even after having read the whole paper (particularly line 343) and the supplementary materials, it's still not clear to me what the OR of 3.05 represents. The authors say in the abstract that rural birthplace together with town residence carries the highest TB risk, but this doesn't look quite consistent with Figure 5B (or at least, TB risk is similarly high in town resident regardless of their birthplace). Also, from

the way it's written, the reader might assume the 3.05 corresponds to this "rural birthplace, town residence" category, but in Table B it seems to actually refer to the "town birthplace, town residence" category. The authors refer also in the abstract to a significant interaction between education and age, but without providing further explanation.

We have revised our reporting of the effects in the Abstract as below. For male gender and town residence we believe the OR are interpretable as the alternate category is implied (and is further described in the Methods).

"We find that factors commonly identified in other global populations tend to replicate in our study, e.g. male gender and residence in a town had significant effects on TB risk (OR: 3.02 [95% CI: 2.30-4.71]; OR: 3.20 [95% CI: 2.26-4.55]). We also tested for demographic factors that may uniquely reflect the historical changes in health conditions in South Africa. We find that socioeconomic status (SES) significantly interacts with an individual's age (p=0.0005). We further find that being born in a rural area and moving to a town strongly increases TB risk, while town birthplace and current rural residence is protective."

For the description of the interaction effects we agree that the 3.05 OR is confusing. To clarify: "rural birthplace together with town residence carries high TB risk" meant that this category has higher odds of active TB than the rural birthplace: rural residence group and the town birthplace: rural residence group. It is not significantly different from town birthplace: town residence group.

To break down the interaction effect, we set rural birthplace: rural residence group as the baseline group and manually calculated contrasts for the other three groups. We explain this in the results as " We found that lifelong town dwellers had about twice the odds of active TB (OR = 2.16 [1.43-3.28], p< 0.001) than the baseline group. Individuals born in a rural area and currently residing in a town had similar outcomes as lifelong town dwellers (OR=2.19 [1.14 -4.20], p= 0.018). Taken together, these show that town residence increases risk regardless of birthplace. Interestingly, individuals who were born in a town and later moved to rural areas are even more protected than individuals born and currently residing in rural areas (0.33[0.16-0.71], p =0.004) "

Please find the revised Figure 5 below for your verification. We interpret this as current/recent residence in a town is likely driving increased exposure to TB, especially during adulthood as rural birthplace appears to have no protective effect on risk.

We have changed our abstract text to state "We find that identified two interactions: age by socioeconomic status (SES) significantly interacts with an individual's age (p=0.0005) indicating that protective effect of higher SES changed across age cohorts. ". In the discussion we discuss this interaction more; " Higher SES increasing TB risk at older ages is contrary to findings in populations in the United States and Mexico (61). This unique pattern may reflect South Africa's recent history of Apartheid and post-Apartheid societal and economic shifts. During Apartheid, individuals from historically marginalized backgrounds had limited career options, but some were able to become teachers, police officers, or nurses. Such occupations are associated with higher education

requirements and would have facilitated access to larger salaries, transportation, and mobility; potentially leading to better diagnosis and treatment. Alternatively, the observed pattern of our interaction effect at older ages could be explained by survivor bias or our operationalization of SES in this study. Highest completed level of education (e.g., grade, diploma, degree, etc.) is a blunt measure of SES, and does not fully capture all SES facets, including social, economic, and cultural capital (63,64), and universal access to education increased post-Apartheid (65). Additionally, we only sample from community health clinics, not private clinics, thereby missing a fraction of the SES spectrum.
"

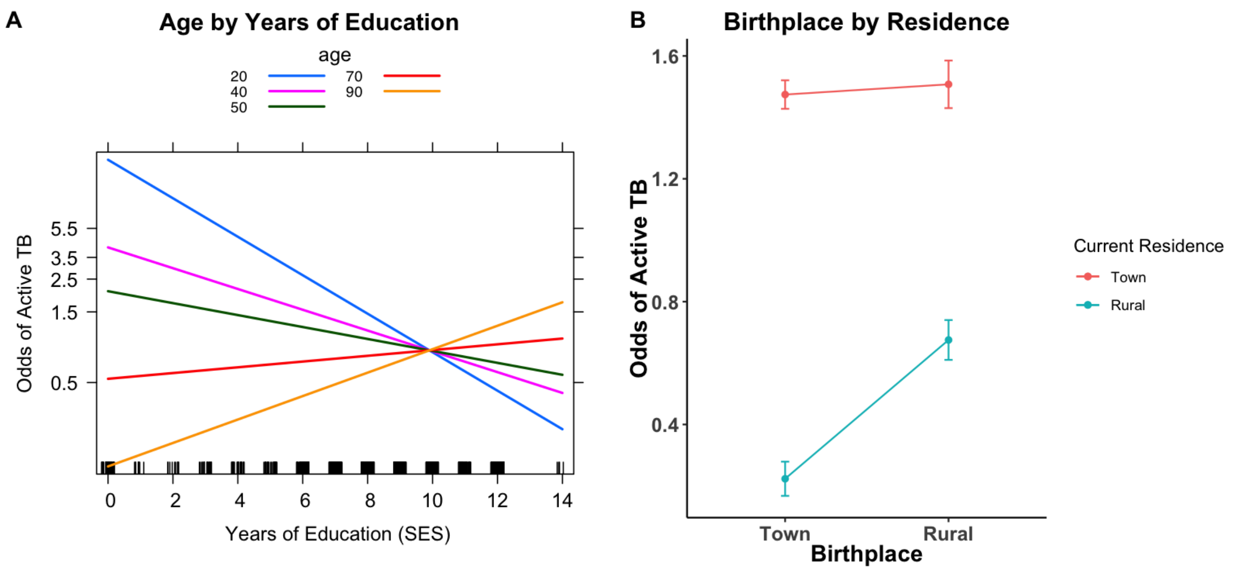

Line 62: "59% of TB patients on a TB programme" – which programme? Can the authors provide a citation?

Thank you for the comment, we have edited the text to "59% of TB patients on the National TB programme" and provided an in-text citation: South African National Department of Health, South African Medical Research Council;, Human Sciences Research Council, National Institute for Communicable Diseases;, World Health Organization, United States Agency for International Development, et al. The First National TB Prevalence Survey | South Africa 2018 [Internet]. 2018

Line 74: Should be "more than household", not "moreover than household".

- **We revised this sentence accordingly, "In South Africa, TB transmission is driven largely by community spread than by household contacts (10,11)"**

The third paragraph of the Introduction is quite weak – it's not clear what the authors are trying to say here. I'd recommend removing this or else reworking it to make it clearer. This seems to relate to the second sentence of the Discussion (lines 369-371), which I also didn't understand. The authors refer to "population-based controls", but they're not population-based (the controls

are recruited from health facilities). And even if they were population-based, how does that make them "useful", and how are they "better" than other strategies for obtaining controls? I would probably remove this sentence too.

- **We have revised this section to clarify that the TB epidemiology of South Africa affords this study a high level of certainty that a majority of our controls have been exposed to Mtb, leading to similar statistical benefits to population-based controls from endemic populations, that is, similar disease exposure to cases. While the controls are not sampled randomly within the community (but rather individuals testing negative at the public health clinics), the definition of a clinical control for TB is somewhat more stringent than this. There is an expectation that controls would be positive for exposure via TST/IGRA testing and negative for active TB via GeneExpert and chest X-ray. We are not able to meet all of these criteria in South Africa; although IGRA positivity from this community is 87% (n= 61/70 controls). Hence, we prefer not to call them 'clinical controls' and feel that a population-based control definition is more accurate for our sample. Please see below the revised text from the introduction:**
    - **"In case-control studies, controls should have similar disease exposure profiles to the cases. Population-based controls risk differential disease exposure, a concern in low-incidence populations that can bias statistical associations. However, in high-incidence populations with well-characterized disease burdens and transmission, population controls can greatly improve statistical power (9). In South Africa, TB transmission is driven largely by community spread rather than household contacts (10,11) and TB latency increases with age, reaching 80% by age 30 (12–15). These epidemiological conditions help ensure our cases and controls have approximate disease exposure profiles. In this context, controls from our study sample are people with suspected TB from local health clinics who were microbiologically confirmed to not have active TB. Controls are assumed to either have been previously exposed to or infected with Mycobacterium tuberculosis (i.e., LTBI). Cases, in contrast, are people who have microbiologically confirmed active TB or self-report a past active TB episode."**
    - **And from the Discussion: "In this study, we demonstrate the utility of study designs other than household contacts, such as using population-based controls when disease exposure is extensive and transmission is community-spread (Munch et al., 2003) as seen in other studies in low-resource, high-burden countries (Duchen et al., 2023; Lienhardt et al., 2005)"**

Line 116: Why are the dates underlined?

- **We have now removed the underlines from the date, thank you for your comment.**

Line 128: Is “clinically confirmed” the correct term here? Do the authors mean microbiologically confirmed?

- **We prefer to use the term, “clinically confirmed” here because patients are screened based on their symptoms prior to a microbiological test; only once a nurse has assessed them as having symptoms consistent with TB do they take a GeneXpert test. Following the use of the term, “clinically confirmed”, we have the following sentence: “GeneXpert results, diagnostic test date, TB strain (drug resistance), and TB medication regimen were used to validate clinically confirmed progression to active TB.”**

Line 138: “relies on population-wide TB exposure” could perhaps be better written as “assumes population-wide TB exposure”. The authors spend a lot of time explaining why they didn’t use IGRA/TST, but I’m not sure this is relevant – could this be omitted?

- **IGRA/TST is considered a gold standard for classifying controls, indicating that study controls have Mtb infection (LTBI) or have been exposed to Mtb. In our NIH grant applications, reviewers have typically expected this validation for our controls, however, their specificity (ability to determine a negative result is truly negative, that is, a negative IGRA/TST result means that person never was exposed to Mtb) is compromised in high incidence populations and where a majority of the population have received a BCG vaccine (resulting in a positive TST result irrelevant of Mtb exposure). Therefore, to increase our sample size and maximize power we defer to the several studies that have shown population-wide exposure to Mtb in South Africa. We feel compelled to explain this logic in this study since the study design standard in low incidence populations is to sample from household contacts (with known/assumed Mtb exposure) and IGRA/TST testing.**

Line 204: “Residing in an urban or rural environment is an established risk factor for TB status.” Please provide citations to support this statement. The authors later cite a number of relevant studies in the Discussion [53-56], but two of these studies are from Iran, one is from Tanzania and only one is from South Africa. The South African study [55] did not show the TB risk to be higher in urban settings (as the study was only done in an urban setting, with no rural comparison).

- **We now provide several additional papers that establish that residence i.e urban or rural environments are TB risk factors : “Residing in an urban or rural environment is an established risk factor for TB status (Chadha et al., 2005; Davies, 2005; de Vries et al., 2010; J. P. Smith et al., 2022) and was included in our common risk factor model, as above. Previously, we have shown that migration from an individual’s natal town has increased over the past two generations in the rural Northern Cape Province (M. H. Smith et al., 2023). In addition, a recent longitudinal study leveraging data from South Africa’s National Health Laboratory Service showed that incorporating cross-municipality migration improves the ability to predict TB incidence (Fofana et al., 2023). For our “residence model”, we hypothesized that**

migrating from a rural to urban area in one's lifetime increases the odds of active TB status due to greater exposure to Mtb. Here, we include an interaction between current residence and birthplace in the common risk factor model. Setting this interaction allows us to examine four patterns, namely: lifetime rural residence, rural birthplace to urban residence, urban birthplace to rural residence, and lifetime urban residence"

Line 253: Where the authors say that 13% of people in the census had completed primary education, they're presumably focusing on adults aged 18+, to be consistent with the age range sampled in this study? Maybe say "13% of adults" to avoid confusion.

Thank you, we have adjusted the text to say "13% of adults".

Line 260: "To our knowledge, this is the first study to report ancestry proportions of a clinical population in the Northern Cape Province, South Africa." If you're going to keep the ancestry data in the paper, I believe this sentence should go in the discussion, not the results.

- **Thank you for your comment we have now moved this to the discussion**

Lines 275-280: I'd put the explanation of the term "coloured" in the Methods section (maybe under Study covariates) rather than the Results section.

Thank you for the suggestion. Because the term "coloured" is unfamiliar with an American / European audience or may suggest negative racial connotations, we would like to clarify its usage in the first instance of Results. We are worried that if we put the explanation in the Methods section, readers could overlook the description and react negatively. The use of this term in South Africa is common, but also sensitive. We requested a consultation from the Stellenbosch University Health Research ethics committee to discuss ethical concerns around our findings as presented in the manuscript and this was one potential issue that they highlighted as prone to misunderstanding.

Lines 291-293: This is repeating what was already stated in the Methods section.

We have now removed the repetitive statement.

Figure 4 seems a bit unnecessary – it is mostly just repeating the information in Table 1?

We have now moved Figure 4 to the Supplementary Text.

Line 319: Why do the authors say SES had no effect when the p value is 0.03?

In the previous version of the analysis, 0.03 did not pass the Bonferroni corrected threshold. In our revised version of the Common risk factor model the exact p-value is 0.006 and passes the Bonferroni corrected threshold of 0.0825. We have added a sentence of this new results

Lines 321-2: I don't think it's correct to describe the relationship as "U-shaped". That would imply that (for a given age) there is a very non-linear relationship between education and TB risk, which is not what the authors are showing in Figure 5.

We removed the description, "U-shaped", and instead focused on the interaction and protective/risk of SES at different ages.

"The effect is such that higher SES at younger ages (18-39 years old) is protective against TB, and higher SES at older ages (>59 years) increases risk (Fig. 5). Middle-aged individuals (40-59 years old) show no relationship between age and SES on TB risk (Fig. 5)."

Lines 350-355: The explanation of random forest modeling belongs in the Methods section, not the Results.

We agree with this comment and have moved this explanation to the methods and supplementary methods

Lines 357-8: "The random forest model also stratified the variable importance by cases and controls." This doesn't make sense to me (though I admit it's not my field of expertise). The outcome is a binary indicator so how can the predictors for cases be different from the predictors for controls?

We agree that the initial result was confusing. We have rerun our Random forest analyses and report results without any stratification.

Lines 405-409: I didn't follow the rationale. Even if migration patterns have changed over time, why does this imply it needs to be investigated as a TB risk factor?

- **We are sorry this was unclear. Because present residence in a town (relative to a rural village) was the strongest predictor of active TB and we know that migration patterns have changed over the past couple of generations, we wanted to investigate the effect of migration. Birthplace was the only other factor that we collected in the survey that would speak to this effect. That is, does the approximate length of time spent in a rural or peri-urban area impact risk, or is present residence the important variable.**
- **We also reference this in the "Hypotheses and Models of Progression to Active TB" section in our paper: In addition, a recent longitudinal study leveraging data from South Africa's National Health Laboratory Service showed that incorporating cross-municipality migration improves the ability to predict TB incidence (58).**
