# Supplementary material for: Strong effect of demographic changes on Tuberculosis susceptibility in South Africa
Source: PLOS Glob Public Health. 2024 Jul 23;4(7):e0002643. doi: 10.1371/journal.pgph.0002643 (PMC11265723; doi:10.1371/journal.pgph.0002643)
Supplement: Attachment — Submitted filename: Response to Reviewers2.pdf [file pgph.0002643.s005.pdf]

Dear Dr. Govender,

We have revised our manuscript entitled "Strong Effect of Demographic Changes on Tuberculosis Susceptibility in South Africa" in response to two reviewers. Our responses are shown under each comment in blue text. In order to understand how underlying population structure (differences in genetic ancestry across towns) might affect our model results, we have performed additional exploratory analysis in response to Reviewer 1. As this analysis reflects a subset of the data, results are primarily included in the Supplement. Regarding the semantics of "controls", we have removed the descriptor of 'population-based' from the manuscript. Please note, that this in no way affects the model results or indeed the discussion of any potential biases associated with study design. Please find a revised discussion for in-depth clarification. Finally, the genetic SNP array data is currently in the process of being deposited in the European Genome Archive. We hope these modifications meets the high standards of PLoS Global Public Health. Please do not hesitate to contact us with questions.

Sincerely,

Profs. Brenna Henn and Marlo Moller, *co-corresponding authors

>>

Reviewer 1:

The authors have done a thorough job of addressing my comments. I appreciate the attention to detail and found the responses to be generally robust. I have two minor outstanding comments:

Line 219 - Please justify the use of the particular populations included as reference groups in the ADMIXTURE analyses. Also why was k=5 chosen? Were other values of k tested?

- *We have previously analyzed genetic ancestry data for other populations from the Western and Northern Cape (e.g. Uren et al 2016, Genetics; Petersen et al. 2013, PLoS Genetics; De Wit et al 2010, Human Genetics). Based on the observation of ancestry proportions we know that there are two ancestries (eastern Asian: Indonesian, Malay, Chinese; southern Asian: Sri Lanka, India, Bangladesh) which are minority ancestries typically less than 5% derived from the VOC slave trade at lower fractions compared to Cape Town. European migration is well documented historically and in interviews. Interviews also suggest that recent migration from Bantu-speakers impacts the communities, including Xhosa, Tswana and others. Finally, the Khoe-San ancestry could have been broken down into 2 clusters based on some differentiation between Khoekhoe and San-derived demographic histories. But this ancestry differentiation estimate tends to be unstable in ADMIXTURE, likely due to small samples sizes for the Khoekhoe. Hence, k=5.*

Additional reviewer response: Got it. Please incorporate this information into the methods so it clear to all readers.

Thank you, all this information is now included in the Methods section.

Are there any relatives in your study? Or population structure? If so, please consider linear mixed effects models that control for pairwise genetic relatedness and/or principal components of genetic variation. Since you have genetic data, I'm not sure why it isn't used for data analysis either to allow for modeling of a key covariate and/or to examine ancestry effects on susceptibility.

- *We do see moderate population structure across clinics. Please see Supplementary section "Genetic ancestry and ethnicity" for detailed discussion.*
- *We agree it would be appropriate to include PC covariates or GRM for this analysis. It is the goal of this project is to perform these association analyses in the future with more data, however, we have only genotyped ~159 samples, which will be underpowered to identify genetic associations. Grant applications to genotype the full dataset have not yet been funded and would total ~$60,000 prohibiting us from including them in the current paper.*

Additional reviewer 1 response: Thanks, that makes sense and I understand the financial limitations for genotyping the whole cohort. However, 159 is a reasonable sample size for checking whether your scientific conclusions are likely to change when you account for population structure, which you demonstrate is pretty substantial. For example, I think it would be worthwhile to check if effect sizes dramatically change in magnitude or direction when you include PCs in the model using the reduced dataset. Or potentially you could ask about average TB patterns as a function of average per-clinic admixture proportions. Again, given that you do see population structure, I think it's worthwhile to try to get some handle on how it could be impacting/confounding current conclusions, even though I understand larger datasets would be more ideal to really dig into this.

- Thank you for your comment. We have performed additional analyses to investigate if our conclusions are likely to change after accounting for population structure. To do this, we included the top 10 genetic principal components into our models for a much reduced dataset of 159 individuals. Specifically, we are only interested in the magnitude and direction but not statistical significance since adding 10 extra variables to such a small dataset led to all variables to be statistically insignificant. The revised regression models for the genotyped subset can be found in **Supplementary File 2**. Briefly, the relative importance of the variables is the same after correcting for the top 10 genetic PCs. Town residence and male gender remain important risk factors. No significant OR shifts were observed for the common risk factor or SES models. The residence model showed an attenuation of the 'residence' and 'birthplace' variables. After PC correction, the individuals born in a town and currently residing in a rural area do not have significantly lower odds of Active TB compared to lifetime rural residents. Current town residents, regardless of birthplace, still have the higher odds of active TB compared to current rural residents. As genetic ancestry varies by present residence (Figure 3), incorporating the PCs may suggest that host genetic susceptibility (which covaries with geographic location) is contributing to differential odds of TB. However, while intriguing, the sample size is so small and potentially correlated with other variables, that we hesitate to

expand on this in the main text. We have included forest and interaction plots in **Fig IV in S1 Text**.

Reviewer #2:

The authors have made significant attempts at address the concerns raised around the first submission, and I appreciate that a number of aspects of the paper are now clearer. I also appreciate that they have removed the HIV-positive individuals from the controls (to be consistent with the definition of cases). Unfortunately, though, there are still a number of significant problems.

Firstly, the authors insist on describing their controls as population-based controls, which is simply not true. The controls were recruited from health facilities and were "TB suspects", which presumably means they had symptoms suggestive of TB. This strongly suggests the controls are NOT representative of the general population. Failure to acknowledge this issue leads to problems in the interpretation of the results.

We have now refrained from using the term "population-based" to describe our controls. We have revised our description of our controls in the discussion:

"Controls from our study sample are people with suspected TB from local health clinics who were microbiologically confirmed to be negative for active TB. Controls are assumed to either have been previously exposed to or infected with M.tb (i.e., LTBI). In South Africa, TB transmission is driven largely by community spread, rather than household contacts (28,29)."

The paragraph in lines 91-102 is still confusing and unnecessary.

We have deleted lines 91-102.

As I commented in the original submission, older people are more likely to attend clinics than younger people, and thus one cannot assume the age distribution of the controls to be the same as the age distribution of the general adult population. This makes it dangerous to infer a survivor bias based on the difference in age distribution between cases and controls.

Thank you for your feedback. One clarification is that we are not comfortable with the statement that older individuals in our catchment are more likely to attend community health clinics compared to younger people. For example, older individuals are less likely to have transportation to the clinic, and often have childcare duties which prevent them from leaving home. It is possible that older individuals, because of immunosenescence, are more likely to have symptoms that overlap with TB and there are screened more often than younger ages. Regardless, this is rather an empirical question and would require a survey of both the clinical and general population.

In your original submission comment you wrote: "*The interpretation of Figure 2 could be questioned. The authors interpret the (relatively) low TB risk at older ages as indicating a*

*"survivor bias". Although that is possible, one should also consider that (a) these are self-reported data that could be affected by recall bias (more important in older adults), and (b) these are data from patients attending health facilities, and not a population-based sample (one might expect that older adults would be more likely to be attending health facilities because of chronic conditions). I did also wonder whether it was really necessary to include Figure 2 in the paper (and if so, whether it should not go in the Results section, rather than the Methods section).***"**

Previously, you acknowledge that survivor bias is a possibility**,** but also suggest that the empirical odds patterns are based on an unrepresentative sample of the general population (sample-selection bias). We agree with you. Indeed, both survivor- and sample-selection bias are forms of selection bias. Generally, selection bias is difficult to predict, measure, and mitigate. Despite the inherent challenges in measuring these types of bias, we attempt to do so by calculating empirical odds of active TB. Our intention here is to show that bias (regardless of what mode) may potentially drive some unexpected patterns we see in our downstream analyses such as our Age by education interaction effect. To clarify this, **we have added this paragraph in the discussion:** "*We considered whether our sampling strategy displayed any indication of selection bias. We hypothesized that the risk of active TB should monotonically increase with age, reflecting cumulative lifetime exposure to the M.tb pathogen. Contrary to this, our findings revealed a non-monotonic relationship. Starting with the youngest age group (18-28 years), the empirical odds of active TB increased with age, peaking in the 29-38-year-old age group, followed by a progressive decline in empirical odds till the oldest age group (79-88 years) (Fig II in S1 Text). This unexpected pattern can be indicative of selection bias, possibly driven by survivor bias —where individuals dying from TB are absent from the study population at older age bins and/or related to whether our controls are representative of the general population (sample-selection bias). Generally, selection bias is difficult to measure and mitigate (54–56), especially in case-control studies where controls are recruited from a clinical setting. Recall bias in older adults could also lead to the lower observed empirical OR in older age bins; however, because TB treatment is a six-month course, and incomplete treatment regimens lead to relapse or life-threatening drug resistant TB, it is generally a life event that people remember with the exception of pediatric TB."*

In addition, we restructured Figure 2 to just show density plots of age and years of education, and made the empirical odds plot a separate figure in the supplemental text. The density plot of Figure 2A, although containing the raw data from which the empirical odds plot is computed, is simply meant to be a visualization of the continuous age distribution. This is now better referenced as such in the text, and we apologize for omission. We write: *"Cases and controls had similar mean ages, 43.6 and 43.1 years respectively (Wilcoxon rank sum p-value = 0.959) (Fig 2A). We found a significant difference in SES between cases and controls, with mean of 7.7 years and 8.3 years, respectively (Wilcoxon rank sum p-value = 0.0019) (Fig 2B)."*

Secondly, the authors still are not providing a clear motivation for why the genetic data belong in this study. The data don't say anything about TB risk, so don't they belong in a separate paper?

- Please refer to our earlier response to Reviewer 1 in the last rebuttal, as well as the current requests by Reviewer 1 in this reply.

Thirdly, the authors have only partially addressed my comment about the unexpected finding that TB risk is lowest in people who are born in towns but migrated to rural areas. It's unsatisfactory that the authors rely on hypotheses of clinic staff that are neither logical nor supported by evidence. If they really wanted to document the perspectives of clinic staff (as they say in their response letter), I would have expected a related research objective with an appropriate qualitative research methodology.

- In line with and in the spirit of PLoS's policy on inclusivity in global research (https://theplosblog.plos.org/2021/09/announcing-a-new-plos-policy-on-inclusion-in-global-research/), which we thoroughly agree with, we include in the manuscript the feedback we received from the community during our return of results. This is a very small, yet, important step towards "de-colonizing" science and increasing transparency. We agree that the clinics' staff hypothesis may not be supported by evidence due the waning effect of BCG by adulthood, but it's still their voice, and as a historically under-represented community (in science and government – throughout colonialism and Apartheid in South Africa), we feel it is important to include it. We have rephrased this section to be clearer that it is one of a couple possible explanations, and we are reporting this as feedback from the community.

Fourthly, the authors have removed from the main text the first statistical model (the "Common risk factor model", i.e. the model without interactions). It's not clear why this was done – I find it quite difficult to interpret the results of the interaction models without being able to compare against the results of the simpler model without interactions.

- Thank you for the feedback, the "Common risk factor model" table is included as Table 1.

Lastly, there is still a lot of explanation and interpretation in the Results section that should rather have gone in the Methods section or others sections. For example, there is much description of the statistical models and the hypotheses that are being tested, which should have gone in the Methods section. The motivations for both hypotheses are not clearly justified – it is hard to see a clear logic behind them, and the logic is only really explained properly in the Discussion.

- Thank you for your comment. However, previously we received comments that it was hard to interpret the hypotheses and models. Thus, we have moved the motivation to immediately precede the discussion of the models and significantly fleshed out the rationale, rather than being buried in the middle of the *Methods* section. We believe the logic behind these hypotheses continues to stand. As you mention, additional detail can be found in the *Discussion*.

Minor comments

On page 83, the authors mention in their response that IGRA positivity was 87% (61/70) in the controls. This seems like an important result, which I don't remember seeing in the

paper/supplementary materials. Could it be included? How was the subset of controls selected for IGRA testing?

- Correct, this result wasn't in the paper/supplemental materials. We take the reviewer's suggestion and have included this unpublished result in the paper: *"We did not perform IGRA and TST tests for all controls in our cohort because IGRA testing requires blood draws and is often prohibitively expensive for large cohort studies, and TST is not readily available in South Africa. To validate the LTBI rate in our controls, we IGRA-tested (within 3 days of the participant's GeneXpert result), a random selection of our sample controls (n=70) and found that they have an 87% LTBI rate (IGRA+)."*

Lines 211-212: I don't quite understand. I would have thought that if you ran 10 imputations there would be 10 datasets (not 2).

- Thank you for the comment. The parameter *m=2 i*ndicates that we generated two complete imputed datasets that were used for pooling. The parameter *maxit=10* does not refer to the number of imputed datasets but the maximum number of iterations per imputation process within each dataset. In these iterations, the mice function cyclically updates each variable with missing data, refining the imputation by using the current complete data at each step. We have now adjusted our text to say: *"We set the parameters of our imputation using recommended settings (35,36) generating two imputed datasets (m=2) that were run for 10 iterations each (maxit =10). "*

More details of imputation can be found in the in-text citations.

Lines 153-160: I still think this side-note on TST and IGRA testing is not really relevant in the Methods section, and could be either cut or possibly moved to the Discussion. It would be relevant if the authors included the previously mentioned IGRA positivity results in the paper.

- We relocated the limitations of TST and IGRA testing for validating *M.tb* exposure from the methods section to the discussion.
- We have taken the reviewer's suggestion and included our IGRA results for a subset of the sample.
